# A Digital Multigate Doppler Method for High Frequency Ultrasound

**DOI:** 10.3390/s140813348

**Published:** 2014-07-24

**Authors:** Weibao Qiu, Zongying Ye, Yanyan Yu, Yan Chen, Liyang Chi, Peitian Mu, Guofeng Li, Congzhi Wang, Yang Xiao, Jiyan Dai, Lei Sun, Hairong Zheng

**Affiliations:** 1 Paul C. Lauterbur Research Center for Biomedical Imaging, Institute of Biomedical and Health Engineering, Shenzhen Institutes of Advanced Technology, Chinese Academy of Sciences, Shenzhen 518055, China; E-Mails: zy.ye@siat.ac.cn (Z.Y.); ly.chi@siat.ac.cn (L.C.); pt.mu@siat.ac.cn (P.M.); gf.li@siat.ac.cn (G.L.); cz.wang@siat.ac.cn (C.W.); yang.xiao@siat.ac.cn (Y.X.); 2 Department of Electronic Engineering, City University of Hong Kong, Hong Kong, China; E-Mail: yanyanyu2-c@my.cityu.edu.hk; 3 The Hong Kong Polytechnic University Shenzhen Research Institute, Shenzhen 518057, China; E-Mails: ap.cheny@connect.polyu.hk (Y.C.); jiyan.dai@polyu.edu.hk (J.D.); 4 Interdisciplinary Division of Biomedical Engineering, The Hong Kong Polytechnic University, Hong Kong, China; E-Mail: lei.sun@polyu.edu.hk

**Keywords:** flow visualization, multigate PW Doppler, digital quadrature demodulation, high frequency ultrasound

## Abstract

Noninvasive visualization of blood flow with high frequency Doppler ultrasound has been extensively used to assess the morphology and hemodynamics of the microcirculation. A completely digital implementation of multigate pulsed-wave (PW) Doppler method was proposed in this paper for high frequency ultrasound applications. Analog mixer was eliminated by a digital demodulator and the same data acquisition path was shared with traditional B-mode imaging which made the design compact and flexible. Hilbert transform based quadrature demodulation scheme was employed to achieve the multigate Doppler acquisition. A programmable high frequency ultrasound platform was also proposed to facilitate the multigate flow visualization. Experimental results showed good performance of the proposed method. Parabolic velocity gradient inside the vessel and velocity profile with different time slots were acquired to demonstrate the functionality of the multigate Doppler. Slow wall motion was also recorded by the proposed method.

## 1. Introduction

Noninvasive visualization of living tissues with high resolution is an indispensable technique to observe physiological activity on a microscopic scale. The conventional imaging approaches including computed tomography (CT), magnetic resonance imaging (MRI), and ultrasound have been limited to resolution on the order of millimeters. Among the available high resolution imaging modalities (such as micro-CT [1], micro-MRI [2], optical microscope [3], and high frequency ultrasound [4]), high frequency ultrasound has become more acceptable because of a good balance of spatial resolution, penetration depth, cost and safety. It has been extensively employed to achieve microscopic resolution for clinical and preclinical studies. High frequency ultrasound has had a significant impact upon diagnosis of the eye diseases [5], skin tumor [6], and cardiovascular diseases [7], which offering high resolution imaging. Anterior segment of the diseased eye, the inflammatory process of skin tumor, the characterization of the arterial plaque and stenosis in the vessel have been evaluated by the high frequency ultrasound. Currently, much of the knowledge of treatment strategies has been obtained from understanding the physiological and functional mechanism responsible for diseases in animal models (mice, rat, and zebrafish, *etc.*). Preclinical research on small animal models has also been propelled by high frequency ultrasound in different areas including cancer research [8], cardiac research [9], and developmental biology [10].

High frequency (usually >20 MHz) ultrasound is able to delineate small structures with fine spatial resolution on the order of tens of microns [4]. Single element transducer based system provides a viable and cost-effective solution, which is a widely used technique for various biomedical studies [11–13]. Array based high frequency ultrasound system can achieve electronic dynamic focusing to increase the depth of field, which expands more of the applications [14]. A commercial system (Vevo 2100, Visual Sonics, Inc., Toronto, ON, Canada) became available in 2009 [15]. State-of-the-art technology of high frequency phased array transducer was successfully fabricated with very small size (∼3.2 mm × 2 mm, size of active 64 elements) [16], which open a new era in the ultrasound society.

High frequency ultrasound has been used for assessing the morphology and hemodynamics of microcirculation with small size vessels and slowly moving blood flow [17,18]. Detailed information of the blood flow can be achieved by high frequency ultrasound for the study of tissue development and function, as well as in examining disease processes such as atherosclerosis and cancer. Several methods were proposed to measure the velocity gradient of the flow including vector Doppler, speckle velocimetry, particle image velocimetry, and multigate Doppler. Vector Doppler can be used to obtain angle independent velocity gradient information [19], but multiple ultrasonic beams were required. Ultrasound speckle velocimetry was used to acquire fluid velocity by analyzing the speckle signal backscattered from particles moving with the flow [20]. However it required very high concentration of seeding particles and low flow shear. Ultrasonic particle image velocimetry was proposed by identifying and tracking the flow tracer (usually ultrasonic microbubbles) within the flow field [21]. The drawback of this technique was limited scanning time and high cost due to usage of microbubbles. Ultrasonic velocity profile had been successfully measured by multigate Doppler method [22,23]. Spatiotemporal information and efficient flow mapping were achieved for flow measurement in biomedical applications. However, the traditional multigate Doppler method was still based on analog solution which made the implementation complex. A new method should be developed to push forward the multigate Doppler technique, especially for high frequency ultrasound to improve the image quality and reduce the system cost.

Traditionally, the analog demodulator was the first choice for Doppler scanner [24,25]. Electronic components such as mixer, sample-and-hold device (S/H), and low-pass filter (LPF) were involved to achieve the blood flow measurement in medical applications. However, these components not only introduced extra noise, but were also limited to a specific transducer with a given frequency range. A completely digital implementation of Doppler had many advantages, such as low noise, stability, flexibility, and small size. In this paper, a multigate Doppler profiling method was proposed by using Hilbert transform based quadrature demodulation scheme to achieve fast and accurate flow measurement. Slowly moved wall motion pattern was also recorded by the proposed digital multigate Doppler method.

This paper proposed a novel flow measurement method for high frequency ultrasound applications. This paper was organized as follows: the algorithm of digital quadrature demodulation and multigate data acquisition were described in the Methods section. Detailed design of a programmable imaging platform was also presented in this section. Experimental results were described in the Results section.

## 2. Methods

### 2.1. Digital PW Doppler

**Figure 1.**
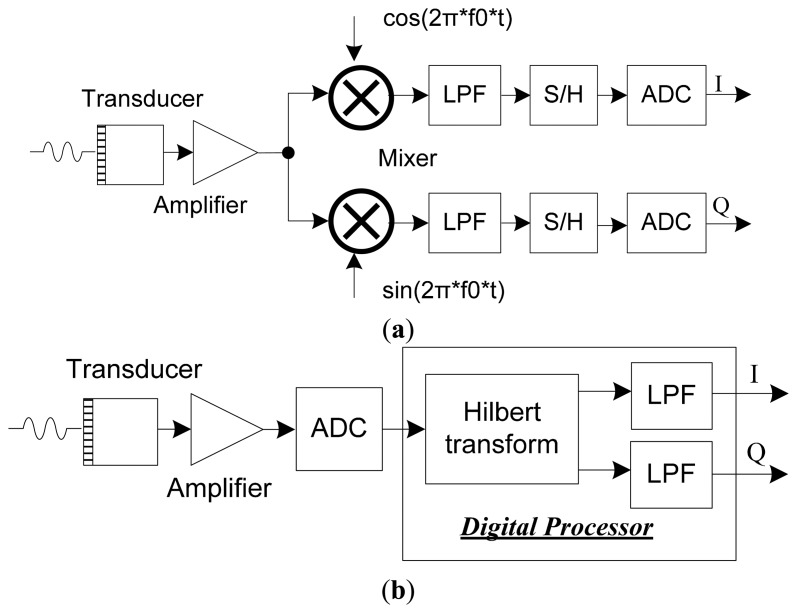
Different schemes for PW Doppler. (**a**) Conventional analog demodulation; (**b**) Hilbert transform based quadrature demodulation method.

The demodulation schemes for PW Doppler imaging method are shown in Figure 1. In terms of traditional analog demodulation method (Figure 1a), the amplified echo signal is demodulated by analog mixer and filter. S/H devices are employed to extract the Doppler information for further process. For digital demodulation method (Figure 1b), the echo signal is acquired directly by analog to digital converter (ADC), which is able to achieve a digital way for Doppler imaging. Analog mixer is eliminated by the digital demodulator scheme and same signal processing path is also shared with traditional B-mode imaging which makes the design compact and flexible. Also digital filter is able to achieve higher signal-to-noise ratio than analog method.

Hilbert transform based quadrature demodulation method is employed to facilitate the PW Doppler acquisition. As an efficient algorithm, Hilbert transform has been employed to process wide band ultrasonic signal [26,27]. The Hilbert transform H[g(t)] of a signal g(t) is defined as:

(1) $$\text{H}\left[\text{g}(\text{t})\right]=\frac{1}{\pi }C\int_{-\infty }^{\infty }\frac{g(\tau )}{t-\tau }d\tau$$

where *C* is the Cauchy principal value of the integral.

Define the input signal as:

(2) x(t)=A(t)*cos[θ(t)]

Taking the Hilbert transform of *x*(*t*) gives Equation (3) according to the Bedrosian theorem [28]. The Fourier spectra of *A*(*t*) is relatively lower than the signal of cos[θ (*t*)], which is not overlapping in the frequency spectrum. Analytic signal can be acquired through Hilbert transform and digital filter which is also shown in Figure 1b:

(3) $$\begin{array}{ll} \text{H}[\text{x}(\text{t})] & \;=\text{H}\left\{A(t)*\cos[\theta (t)]\right\}\\ & =A(t)*\text{H}\left\{\cos[\theta (t)]\right\}\\ & =A(t)*\sin[\theta (t)] \end{array}$$

**Figure 2.**
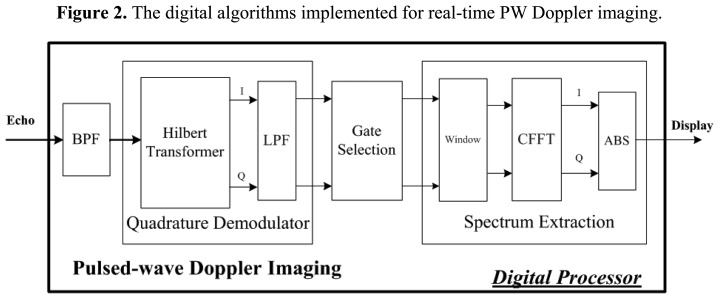
The digital algorithms implemented for real-time PW Doppler imaging.

Figure 2 shows the detailed algorithm achieved for digital PW Doppler imaging. Hilbert transform is employed for quadrature demodulation to process the filtered ultrasonic echo. Gate selection is used to extract the Doppler samples for flow measurement. Spectrum extraction consists of window function (Hamming window in this study), complex fast Fourier transform (CFFT), and envelope extraction. These algorithms are implemented in a digital way for fast calculation and small size.

### 2.2. Multigate Doppler Process

**Figure 3.**
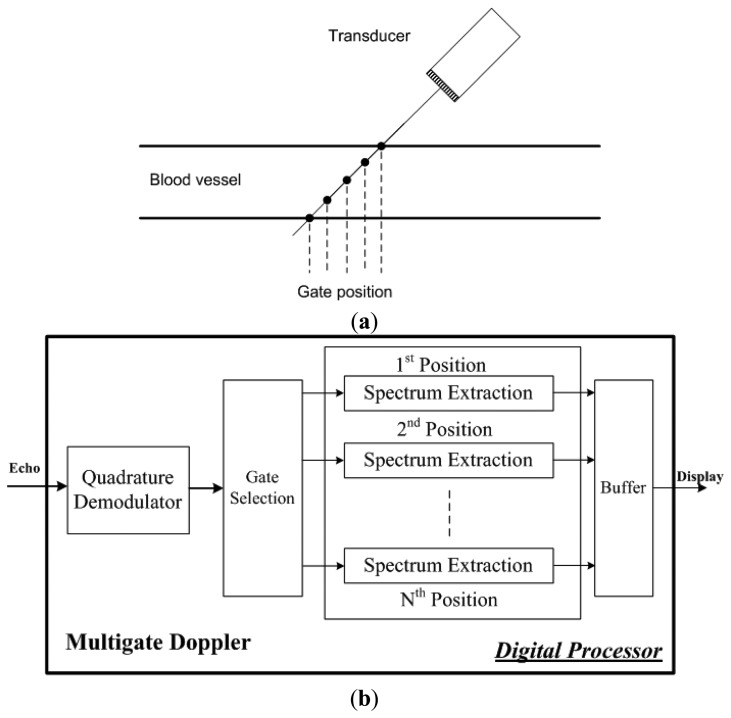
(**a**) Data acquisition scheme for multigate PW Doppler imaging; (**b**) Algorithms of multigate PW Doppler imaging achieved in a digital processor.

Digital implementation of PW Doppler not only offers several advantages over traditional analog method in terms of accuracy, stability and reliability, but also makes it easy to achieve multigate Doppler data acquisition and process. Figure 3a illustrates the scheme of multigate Doppler imaging method. A group of data from different depth of the blood vessel is acquired through different interrogation gate. Velocity profile and gradient in the blood vessel can be acquired by this technique.

Figure 3b shows the algorithms which are implemented in a digital processor for real-time multigate PW Doppler imaging. A quardrature demodulator is used to extract the low frequency Doppler shift from the echo signal. Multiple points are extracted for multigate Doppler measurement. Spectrum extraction is used to calculate the multiple gated Doppler signals.

### 2.3. Imaging Platform

**Figure 4.**
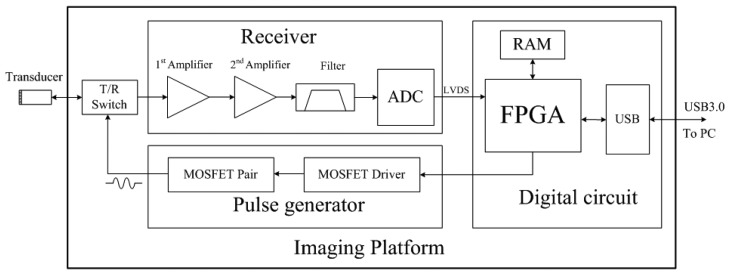
Schematic of the imaging platform for high frequency multigate PW Doppler.

High frequency ultrasound has the ability to measure small size vessels and low speed blood flow, which is an invaluable tool in biomedical applications. An open system was proposed previously in our group for high frequency ultrasound applications [29,30]. In this study, we upgrade the open system with one integrated printed circuit board (PCB) including pulse generator and imaging receiver. The block diagram of newly designed imaging platform is shown in Figure 4. All the electronics are soldered in one PCB for cost-effectiveness and compactness.

Bipolar pulse scheme was used in this platform. The spectrum of bipolar pulse was adjusted to match the bandwidth of transducer for high energy transmission to achieve high signal-to-noise ratio. Two metal-oxide-semiconductor field effect transistor (MOSFET) drivers (EL7158, Intersil Corporation, Milpitas, CA, USA) were employed to accomplish the voltage level shift and high current output to excite the high-speed MOSFET pair (TC6320, Supertex Inc., Sunnyvale, CA, USA). The MOSFET pair could support more than 140 Vpp breakdown voltages and a 2 A output peak current, which are essential for high frequency ultrasound imaging. FPGA is used for frequency control in a digital way through the interface software. The center frequency of the pulse, as well as the number of the pulse were adjustable by FPGA.

In the receiver part, a low noise amplifier (SMA231, Tyco Electronics Co., Berwyn, PA, USA) was used as the pre-amplifier, followed by a low noise differential amplifier (THS4509, Texas Instruments Inc., Dallas, TX, USA) to achieve adequate amplification. A dedicated analog filter was designed for anti-aliasing filtering to remove higher frequency noise. We utilized a high-speed, 12-bit ADC (AD9230, Analog Devices, Canton, MA, USA) with a maximum sampling rate of 250 mega-samples per second (MSPS), which was sufficiently large for the high frequency ultrasound imaging. A high performance FPGA (Cyclone-V 5CGXFC7D7F31C8N, Altera Corporation, San Jose, CA, USA) with great signal integrity was employed to support high speed signal processing, and high programmability. The FPGA had 149.5 K equivalent logic elements and 480 user I/O pins. It included 156 variable-precision digital signal processing blocks, which can efficiently implement various signal processing algorithms. High order filter, Hilbert transform, envelope detection, digital scan conversion, and related algorithms can be implemented. A 2 Gb DDR3 SDRAM (MT41J128M16HA, Micron Technology Inc., Boise, ID, USA) was used for temporary buffering of data. The image data or raw RF data were transferred to a computer through USB3.0 interface.

## 3. Results and Discussion

### 3.1. Platform Implementation

**Figure 5.**
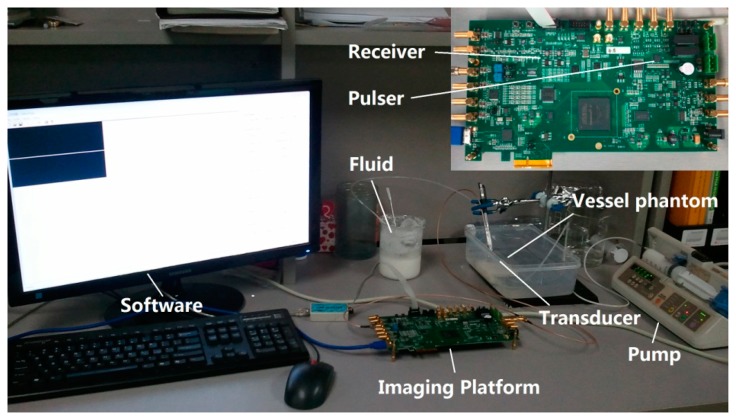
Photograph of integrated imaging platform and experimental setup for multigate PW Doppler measurement.

The prototype of compact imaging platform and experimental setup are shown in Figure 5. The platform is a 12 layers PCB design incorporating high speed pulse generator (programmable bipolar pulse), low-noise frontend electronics (amplifier, filter, *etc.*), high speed ADC (12 bit, 250 MSPS), high speed FPGA (processor for programmable imaging algorithms), and high speed computer interface (USB 3.0 technology). The size of the imaging platform is about 16.8 cm × 10.6 cm. The USB 3.0 interface is designed for high speed data transfer and real time imaging. The interface software for reconfiguration and operation of the imaging platform is designed by Visual C++ language.

### 3.2. Validation Measurement

**Figure 6.**
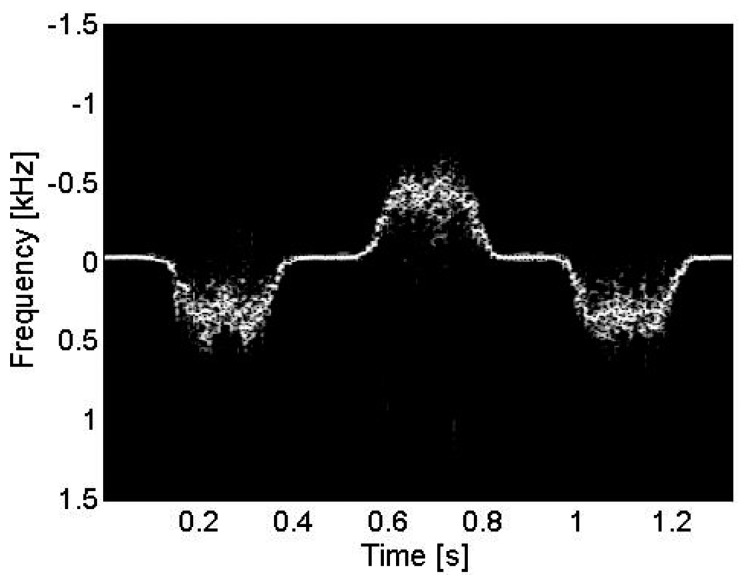
PW Doppler imaging of a moving reflector.

A precision linear motor stage (PLS-85, PI miCos, Eschbach, Germany) was employed to quantitatively verify the accuracy of the velocity measurement by the proposed platform and PW Doppler method. A reflector which was a tissue-mimicking phantom fabricated in our lab [31,32], was put into a water bath container attached on the motor controlled stage. The stage travelled back and forth with the speed of 20 mm/s. A 35 MHz LiNbO_3_ transducer with a focus length of 7.3 mm and 55% bandwidth was employed [33]. The transducer was fixed above the reflector in the container with about 75 degree Doppler angle. A seven-cycle 48 Vpp sinusoidal pulse was generated from the platform with a pulse repetition frequency of 3 kHz. Figure 6 shows the obtained spectrogram, demonstrating the velocity profile of the moving reflector. The dynamic range was set to 50 dB. The measured Doppler waveform agreed well with the appointed velocity pattern after the Doppler angle correction.

### 3.3. Flow Measurement

**Figure 7.**
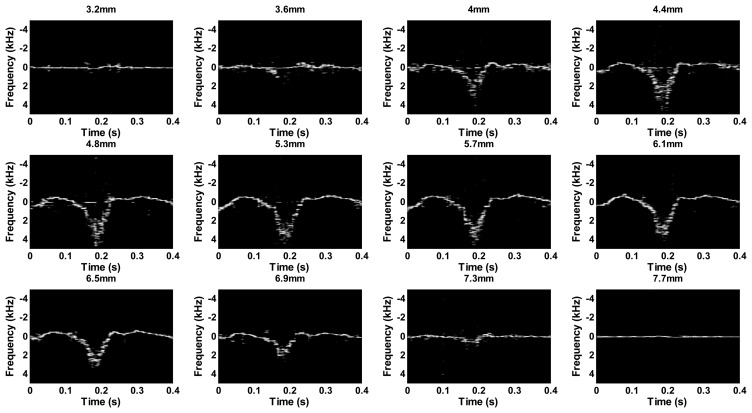
Sonogram of multigate PW Doppler with twelve interrogation gates.

A blood-mimicking fluid was fabricated to test the performance of the multigate PW Doppler measurement. The procedure of the fluid fabrication followed Samavat's method [34]. The fluid was pumped into a vessel phantom, which had a vessel-mimicking hole inside (about 4.5 mm diameter). The experiment setup was shown in Figure 5. The excitation pulse was a seven-cycle 48 Vpp sinusoidal pulse with 10 KHz pulse repetition frequency. The Doppler angle was set to about 75 degree. Figure 7 displays the obtained multigate sonogram of fluid inside the vessel phantom with different interrogation depths. The dynamic range of the sonogram was set to 50 dB. The waveform profiles were changed as increasing the depth. It increased gradually from the edge of the vessel before reaching the maximal point in the center part. Negative waveform profile confirmed that the flow was flowing away the transducer.

**Figure 8.**
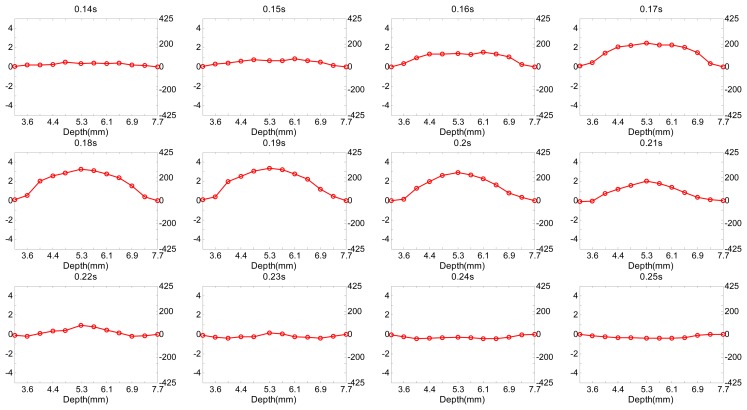
Velocity profile of the flow inside the vessel phantom with different time slots.

Figure 8 shows the velocity profile of the flow in different time. The peak velocities in different depths were extracted from the maximal waveform envelope which was shown in Figure 7. The velocity was low initially and reached maximum value at 0.19 s. A parabolic shape velocity profile can be obliviously acquired by the proposed high frequency multigate Doppler technique.

**Figure 9.**
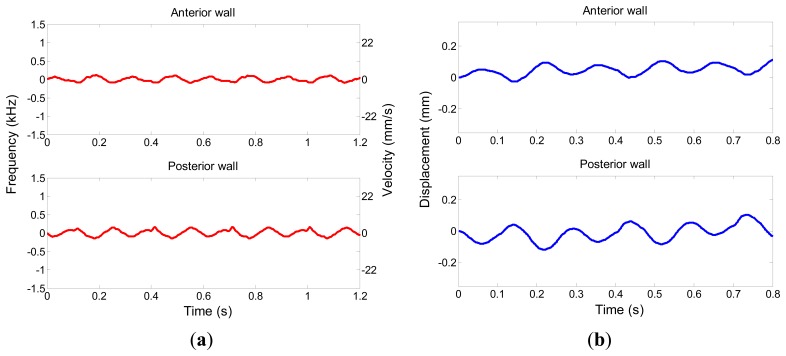
Velocity profiles (**a**) and displacements (**b**) of the anterior and posterior wall motion derived from the multigate Doppler imaging.

Wall motion pattern was also measured by the proposed high frequency multigate Doppler method. The Doppler data of anterior wall and posterior wall were sampled simultaneously. Figure 9a shows the velocity profiles of the anterior wall and posterior wall motion respectively. Figure 9b presents the displacements of the anterior wall and posterior wall calculated from velocity and time. These results demonstrate the feasibility of the slow motion measurement by high frequency ultrasound.

### 4. Conclusions

A completely digital implementation of multigate PW Doppler method was proposed in this paper for high frequency ultrasound applications. Analog mixer was eliminated by a digital demodulator and same data acquisition path can be shared with traditional B-mode imaging which made the design compact, flexible, and cost-effective. A programmable high frequency ultrasound platform by integrating pulse generator and imaging receiver was also proposed to facilitate the multigate Doppler flow visualization. Experimental results showed good performance of the proposed method. Parabolic velocity gradient inside the vessel and velocity profile with different time slots were acquired to demonstrate the functionality of the multigate Doppler. Slow wall motion was also recorded. The proposed digital multigate Doppler method has good potential to be applied in commercial high frequency ultrasound system to facilitate the flow measurement.
